# Colorimetric Detection Based on Localized Surface Plasmon Resonance Optical Characteristics for Sensing of Mercury Using Green-Synthesized Silver Nanoparticles

**DOI:** 10.1155/2020/6026312

**Published:** 2020-01-11

**Authors:** Eman Alzahrani

**Affiliations:** Chemistry Department, Faculty of Science, Taif University, Taif, Saudi Arabia

## Abstract

Development of selective colorimetric detectors that can use green-fabricated silver nanoparticles' (AgNPs) with localized surface plasmon resonances (LSPRs) to rapidly, simply, and selectively detect Hg(II) ions was undertaken in this study. Onion extract was used for synthesising photo-induced green crystalline silver nanoparticles (NPs). The formation of nanoparticles is enhanced when ultrasound irradiation is present; bioligands could serve as stabilizing and reducing agents. Different methods of measurement, including UV-Vis, TEM, SEM/EDAX, FT − IR, and XRD, are effective for characterization of nanoparticles. The spherical nature of green-fabricated AgNPs is confirmed by TEM. High-density, spherical, and uniformly formed silver nanoparticle shapes were found in silver nanoparticle SEM images. The arrangement of AgNPs in the form of face-centered cubic structures was confirmed by XRD patterns. The formation of impurity-free AgNPs was confirmed using the EDAX analysis results. Hg^2+^ with excellent sensitivity was sensitively and selectively detected by employing green-synthesized silver nanoparticles. The reduction of Ag (1) to Ag (0) was confirmed by a slight increase in Hg (II) concentration and progressive reduction of green-synthesized AgNPs, whose absorbance changed abruptly. The reduction of LSPRs by the phosphate buffer medium enables AgNPs to sensitively and selectively detect Hg^2+^ ions by providing good environment. Besides, a selective, sensitive, simple, and rapid method that is proposed for detecting Hg (II) ions in samples of water is presented in the study. Harmful mercury ions in real samples of water (tap and ground water) can colorimetrically and selectively be detected using the AgNPs. The results showed an RSD of below 6% and over 92% of good recovery.

## 1. Introduction

The ecological environment and human health are seriously threatened by toxicity associated with heavy-metal ion contamination [1–4]. Dye, coal, plastic, paper, and natural gas industries exemplify heavy metal pollution sources [5–7]. Because of its highly toxic compounds, mercury ions (Hg^2+^) present a significant concern among numerous heavy metals. Even in low concentrations, intestines, stomach, kidney, heart, and brain can be spoilt by Hg^2+^, which is among the most stable inorganic form of solvated mercuric ions [8–10]. In view of this, close attention should be accorded to selective detection of low concentration mercuric ions in biological systems and waste water; additionally, all contaminated products should be examined to identity the quantity of Hg^2+^. Drinking water should have concentration limits of not more than 2 ppb for mercury ions, as outlined in the Environmental Protection Agency (EPA) and World Health Organization (WHO) requirements [11, 12].

Mercury ion detection can be undertaken using classical methods. Nevertheless, tedious laboratory procedures and expensive instruments complicate the use of such methods. In view of this, there is an increasing need for developing Hg^2+^ ion sensors that could be considered convenient, straightforward, cost-effective, real time, and on-site. Numerous Hg^2+^ ion detection systems, including nanostructures [13, 14], DNA [15], proteins [16], polymers [17–19], and organic compounds [20, 21], have been reported in the literature. Because of the sensitive reaction that metallic nanoparticles have towards the nanoparticle surface local environment, significant attention has recently focused on their chemical sensing applications. Moreover, monitoring is simplified because they exhibit strong absorption or scattering. Because of the unique optical and electronic properties of silver and gold nanoparticles (NPs), they have become suitable for detecting different analytes than other metallic nanoparticles [22, 23]. Development of numerous gold particle- (AuNP−) based colorimetric sensors for detecting Hg^2+^ ions has been undertaken. The area of silver nanoparticle- (AgNP−) based detection system [24, 25] has received less research attention.

Significant research attention in the development of mercury sensors has been accorded to AgNPs, particularly because of soft-soft chemistry involving sulfur with stabilizing ligands on AgNPs surfaces, thus changing the peak position and absorbance intensity, the presence of redox chemistry involving AgNPs (Ag°) and Hg^2+^ leading to formation of Ag − Hg mixture through nanoparticle etching, high sensitivity of AgNPs localized surface plasmon resonances, and cost-effective synthesis [2]. Besides, a UV − Vis spectrophotometer containing specific optical properties and which use LSPRs within the visibility range of 350–800 nm can be used for easy monitoring of AgNPs [26–31]. Radiation chemical reductions [32], photo reduction within reverse micelles [33], and chemical stabilisation with reagents, including sodium borohydride, ascorbate, and sodium citrate [34–37] have been used for synthesizing silver nanoparticles. The aforementioned methods cannot be scaled upwards easily for large-scale nanoparticle fabrication and require toxic chemicals, energy, pressure, or temperature, as well as high costs [38]. Moreover, the use of silver nanoparticles could be limited by the absorption of toxic chemicals on silver nanoparticle surfaces [39]. Biological methods involve the use of fungus [40], enzymes [41], or microorganisms [42, 43]; nevertheless, such methods require special attention to be accorded for preparation of cultures and methods to isolate the fabricated silver nanoparticles [44, 45]. Fabrication of silver nanoparticle using green chemistry would present numerous benefits that include less energy consumption, easy scaling up for large-scale nanoparticle fabrication, high yields, low cost, eliminates the necessity for special preparation of cultures and method of isolation, and environmentally friendly [44, 46–50].

Notably, the use of green-fabricated silver nanoparticles as the colorimeter detector in determining Hg^2+^ ions within phosphate buffer media that suppresses strong interference effects from other ions has received little research attention; in view of this, this study sought to fabricate green AgNPs for sensitive and selective colorimetric Hg^2+^ sensing within phosphate buffer media that suppresses strong interference effects from other ions on the basis of LSPR with green silver nanoparticles acting as the case of the model. To prevent use of hazardous or toxic chemicals, an eco-friendly technique was used for fabricating Ag nanoparticles. Under ultrasonic irradiation, AgNPs were fabricated using extract of onion as the reducing reagent of silver nitrate salt (AgNO_3_). Fourier transform-infrared spectroscopy (FT − IR), XRD analysis, EDAX/SEM analysis, TEM analysis, and UV − visible spectra analysis were used for characterising the materials prepared. On the basis of reduced surface plasmon resonance (SPR) through a UV − Vis spectrophotometer as well as change in visual colour from yellow to colourless, less than 2 minutes could be used for detecting Hg^2+^. Samples of real drinking water were used to perform experiments that had high selectivity to Hg^2+^ ions. Besides, evaluation of the detection method in terms of calibration properties was undertaken.

## 2. Experimental

### 2.1. Chemicals and Reagents

A grocery store in Saudi's city of Taif supplied fresh onions. All chemicals were analytically graded. Acros Organics (Loughborough, UK) supplied the analytical grades of silver nitrate (AgNO_3_) (99.8%) and various metal salts (MnCl_2_, HgCl_2_, CdCl_2_, ZnCl_2_, CuCl_2_, NiCl_2_, CoCl_2_, SrCl_2_, BaCl_2_, CaCl_2_, KCl, NaCl, FeCl_3_, AlCl_3_, and CrCl_3_) employed in the study; no further purification was done on all the aforementioned. The required quantity of salt was mixed in double-distilled water to prepare all metallic salt solutions that were used in the experiments. Moreover, NaOH, HCl, sodium metaborate, boric acid, Na_2_HPO_4_, and NaH_2_PO_4_ were used for preparing 0.1 M buffer solution. No further purification was done to all the chemicals used in the experiment. Distilled water was used for preparing all solutions.

### 2.2. Instrumentation

Fisher Scientific Co. Ltd., from Shanghai, China, supplied the bath sonicator (42 kHz, 100 W). Cambridge Instruments from the United Kingdom supplied a scanning electron microscope (SEM). JEOL Ltd from Welwyn Garden City, UK, supplied the transmission electron microscopy (TEM) instrument. A JEOL JSM 6390 LA analytical device from Tokyo, Japan, was used for conducting the energy dispersive X-ray (EDAX) analysis. Thermo Scientific™ GENESYS 10S from Toronto, Canada, supplied the UV-Vis spectrophotometer. A Bruker diffractometer D8-ADVANCE alongside CuK*α*1 radiation from Coventry, UK, was used for obtaining X − ray diffraction patterns. A PerkinElmer RX FT − IR ×2 device alongside DRIFT attachment and diamond ATR supplied by PerkinElmer from Buckinghamshire, UK, was used for collecting the attenuated total reflectance (ATR) mode.

### 2.3. Fabrication of the Green Ag Nanoparticles

After top skin of the onion was peeled (∼20 gm) and each segment separated, it was placed in room temperatures for 2 days. Filtration was conducted to the new solution to obtain onion extract concoction that is pale white and transparent, whereas the pieces of solid onion were removed, and prepared onion extract was utilised for synthesizing Ag nanoparticles. The concoction of onion extract measuring 20 mL was combined with AgNO_3_ in distilled water (15 mM) to create a total volume of 50 mL with a final AgNO_3_ concentration of 1 mM. The concoctions were placed in the ultrasonic for 50 minutes, which yielded a light-orange colour, an indication that silver nanoparticles had been formed. The concoction was allowed to stay for 3 hours, yielding a deep brown-yellow colour. For removal of excessive free extracts of onion from the concoction, a 15-minute centrifugation was undertaken at 7,000 rpm for dispersions of silver nanoparticles. Finally, double-distilled water used to wash the formed Ag nanoparticles. Overall, the brownish residue is spread within double-distilled water and utilised for additional experiments.

### 2.4. Characterisation of the Green-Fabricated AgNPs

An ultraviolet-visible spectrophotometer was used for monitoring the fabricated silver nanoparticle solution, whereas the silver nanoparticle formation and solution colour were observed using naked eyes. A UV − Vis spectrophotometer was used for measuring the absorbance for the 1 mL sample solution, which was compared against 1 mL distilled water as the blank in the range of 350–800 nm operated at 1 nm resolution.

TEM analysis was used for studying silver nanoparticle formation. Here, 5 *μ*L  sample solutions were placed onto lacey carbon-coated copper grids with a diameter of 3 mm. A Gatan Ultrascan 4000 digital camera connected to a JEOL 2010 transmission electron microscope running at 20 kV was used to obtain TEM images.

SEM was used for classifying silver nanoparticles in terms of morphologies. In the high vacuum mode, 100 pA of probe current alongside 20 kV increasing voltage was used for obtaining images. A 15-minute centrifugation at 1100 rpm was used for separating fabricated silver nanoparticles. The fabricated silver nanoparticles' chemical composition was obtained using the energy dispersive X-ray (EDAX) analysis.

A FT − IR  spectrophotometer with a wavenumber ranging from 4000 to 600 cm^−1^ and 4 cm^−1^ resolution accuracy was used for obtaining a FT − IR spectrum. A ratio of 1 : 100 was used for mixing KBr and the ground sample. Afterwards, clear thin pellets were made after pressing. Recording of the spectra was done in the mode of transmittance as the wavenumber function.

XRD alongside Cu Ka radiation (=1.5405 A) within the 2-theta (2*θ*) range of 35°–80° was used for performing structural analysis and phase identification.

### 2.5. General Procedure for the Colorimetric Determination of Hg^2+^

Generally, the procedure for Hg^2+^ colorimetric determination involved triple dilution of double-distilled deionized water, which yielded a three-fold diluted concentration. The ability of metal ions to detect transition-metal ions (Cd^2+^, Co^2+^, Hg^2+^, Zn^2+^, Cu^2+^, Mn^2+^, and Ni^2+^), alkaline Earth (Ba^2+^, Sr^2+^, and Ca^2+^), representative alkali (K^+^ and Na^+^), trivalent metal ions (Fe^3+^, Al^3+^, and Cr^3+^), and green-synthesized silver nanoparticles of similar concentration (10^−3^ mol L^−1^ and 1 mL), and conditions were investigated by adding 2 mL of three times diluted solution of freshly prepared silver nanoparticle solution and 0.1 M buffer solution measuring 100 *μ*L. Room temperature was used for monitoring the UV − vis  absorption spectra changes and the assays. After 2 minutes of mixing, a digital camera was used for taking photographs.

Indeed, the linear correlation involving the unreacted silver nanoparticles after loading mercury ions followed by the conventional galvanic reaction and the measured intensity of absorption constitutes the premise for the quantitative feedback titration approach. For construction of the calibration curve (*A*_str_*vs*Hg^2+^ volume), the change in conventional absorbance strength at optimum absorption wavelength was measured in form of absorption ratio *A*_str_ denoted by the following equation [51, 52]:

(1) $$\begin{array}{c} A_{\text{str}}=\frac{\left(A^{{}^{\circ}}-A_{\infty }\right)}{A^{{}^{\circ}}}\times 100, \end{array}$$

where *A*° and *A*_*∞*_ denote the maximum absorbance for the absorption band of LSPR (subscripts “°” and “*∞*” represent the blank colloidal suspension prior to injection of required analyte concentration, as well as at infinite duration).

### 2.6. Recovery Experiments

Finally, three separate concentrations of mercury ions spiked with tap and ground water samples were used for performing the recovery experiments. Afterwards, mercury ion concentration in samples was computed using the linear regression equation and the assay response against samples of spiked water. Accordingly, the formula below was used for computing the recovery values [53]:

(2) $$\begin{array}{c} \text{recovery }\left(\%\right)=\frac{\text{calculated }\left[{\text{Hg}}^{2+}\right]}{\text{added }\left[{\text{Hg}}^{2+}\right]}\times 100. \end{array}$$

## 3. Results and Discussion

### 3.1. Formation of Green AgNPs

For use in analytical applications, stable silver nanoparticles should be synthesized against the dilution, over ionic strength, different pH ranges, and long storage period [28, 48, 52]. In this study, wet-chemical green synthesis was used for preparing the green silver nanoparticles because it constituted the most prevalent procedure for fabricating uniform nanoparticles with regulated sizes and strong silver nanoparticles alongside their colloidal dispersions in organic solvents or water [54–56]. The onion extract as an eco-friendly reducing reagent and nontoxic bioextract that reduced silver ions (Ag^+^) to colloidal silver nanoparticles (Ag°) was used for performing the reduction under a sonication bath. In the current study, ultrasonic irradiation was employed at the expense of magnetic stirring as past study reported [51], a significant reduction of Ag^+^ alongside a higher formation of silver nanoparticles when utilising sonication bath, an indication that the reaction rate could be enhanced through ultrasonic irradiation.

### 3.2. Characterisation of the Green AgNPs

#### 3.2.1. Optical Studies

**Figure 1:**
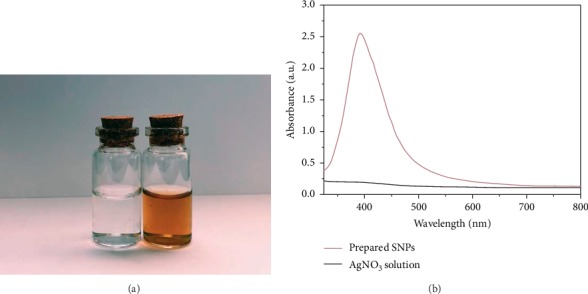
(a) The change in colour of the silver nitrate solution (transparent) after the reduction of silver ions and the formation of silver nanoparticles (dark brown) formed by onion extract; (b) comparison between the UV-visible absorption spectra of the green-prepared AgNPs and AgNO_3_ solution.

Visual observation for silver nanoparticle formation is facilitated when colour change follows the conversion of silver ions to silver nanoparticles. In view of this, silver nanoparticle formation was monitored by visually checking the colour changes, and when the colour of the solution does not change further, the reaction was halted [57–59]. There was a gradual change in reaction mixture's colour from colourless into brown in 50 minutes because of silver nanoparticle formation within the solution, as illustrated in Figure 1(a). This is because of excitation of surface plasmon vibrations in AgNPs [60].

The AgNO_3_ solution and the green-fabricated silver nanoparticles in terms of the UV − Vis spectra are illustrated in Figure 1(b). The results showed that the AgNO_3_ solution had no absorbance, while the silver nanoparticles had an absorbance peak. A narrow, symmetrically sharp, and single LSPR band contained the colloidal solution's absorption spectra. The silver nanoparticles had an optimum absorbance peak of 405 nm, thus implying the presence of a slight-blue change as opposed to the optimum absorbance of between 410 and 422 nm for silver nanoparticles [61–63]. Thus far, the change characterising silver nanoparticle bands lacks a general principle. Nevertheless, dielectric environment, shape, and size differences could be the cause of band shift [64–67]. The final conversion of AgNO_3_solution into AgNPs reached 100% in 50 minutes since the maximum absorbance of the formed AgNPs were not increased. After one month, the fabricated silver nanoparticles remain unaffected, an indication of stability and uniform dispersal for green-fabricated silver nanoparticles within aqueous solution. Besides, the green-prepared silver nanoparticles could be preserved in form of lyophilized powder over a prolonged period without the LSPR property shifting [68].

#### 3.2.2. Morphological Characterisation

**Figure 2:**
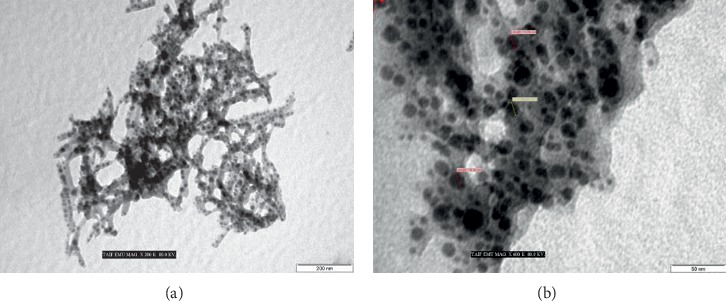
TEM images of the green-prepared AgNPs using different magnifications.

In this study, TEM analysis that can account for fabricated nanoparticle size and morphology was used for characterising green-fabricated silver nanoparticles [28, 69, 70]. Different magnifications for green-fabricated silver nanoparticle TEM micrographs are illustrated in Figure 2. The findings showed that the silver nanoparticles were within the nanorange with spherical shape and with good dispersal and without aggregation. In addition, a thin capping material layer from onion extract on the surface of AgNPs was observed, which can help stabilize the AgNPs in the solution for long period [71].

**Figure 3:**
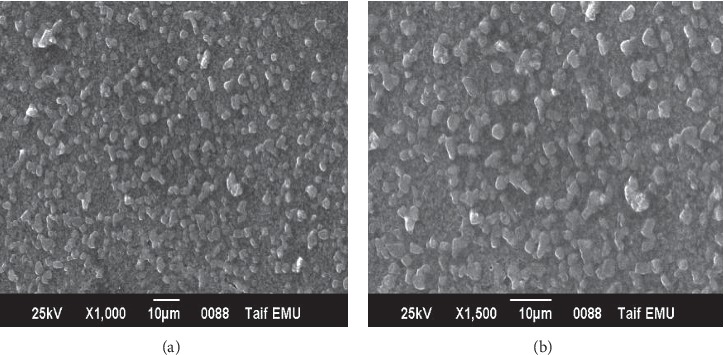
SEM images of the green-prepared AgNPs using different magnifications.

The green silver nanoparticles suspended in sterilized distilled water were utilised for SEM analysis through fabrication of suspension drops onto clean electric stubs, and the water was left to evaporate completely. The silver nanoparticles' SEM image indicated uniformly shaped and spherical nanoparticle formation with onion extract synthesizing high-density silver nanoparticles, a further confirmation of existing monodispersed silver nanoparticles, as illustrated in Figure 3. The same result was obtained by Jae Song and Beom Kim [60].

#### 3.2.3. EDAX Analysis

**Figure 4:**
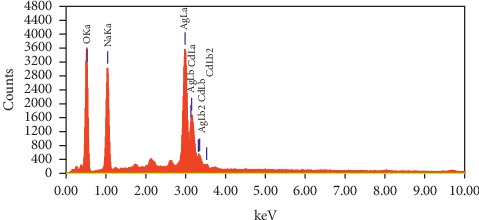
EDAX characteristic spectrum of the green-prepared AgNPs.

Quantitative and qualitative data for fabricated material elements could be given through EDAX analysis. In view of this, EDAX analysis was used for green-fabricated silver nanoparticles. The sample's elemental composition is shown by the EDAX spectrum in Figure 4. Due to surface plasmon resonance, an optical absorption peak at 3 keV, and peaks at between 2 keV and 4 keV, associated with silver's properties lines L and K [60, 72, 73] was found. This showed the presence of silver in the nanostructure. In addition, other aspects could be seen on the left side of EDAX spectrum, that is, sodium (Na) at 1.041 keV and oxygen (O) at 0.525 keV. The tested samples sodium and oxygen peaks appeared from the biomolecules, which are bound to silver nanoparticle surface, and created a thin capping material layer and had stability in solution because of capping materials on the nanoparticle surface. For other groups, a similar outcome was obtained [74, 75].

The quantitative analysis was conducted using the obtained EDAX spectrum. The results showed that oxygen and sodium contents were 35.93% and 23.98%, whereas silver content was high at 40.09% within the samples examined. The EDAX analysis results revealed formation of pure silver nanoparticles.

#### 3.2.4. XRD Analysis

**Figure 5:**
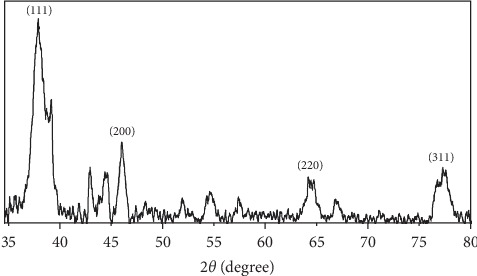
XRD pattern of the green-prepared AgNPs.

X-ray diffraction was used for analyzing the crystallinity of synthesized silver nanoparticles. XRD diffraction peaks of 76°, 64°, 46°, and 38° that correspond to crystal facets for (3 1 1), (2 2 0), (2 0 0), and (1 1 1) were found, as illustrated in Figure 5. There is significant concurrence between face-centred cubic (FCC) structure [76, 77] Ag crystal and the peaks, an indication of crystalline silver presence in silver nanoparticles. Diffraction peaks that corresponded to the precursors (AgNO_3_) or by-products (for instance, silver oxide) did not exist, thus confirming that in situ formation of metallic silver was only possible through reaction of onion extracts. The high crystallinity level for the synthesized silver nanoparticles is reflected by the peak intensity. Nevertheless, the breadth of diffraction peaks is an indication of small sizes of crystallite. This is consistent with the findings of Kumar et al. [78], Balavigneswaran et al [79], and Sheny et al. [80].

#### 3.2.5. FT − IR Measurement

**Figure 6:**
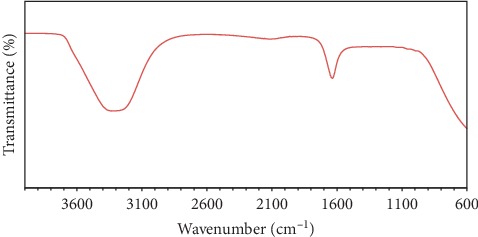
FT-IR transmittance spectrum of the green-prepared AgNPs.

The green-fabricated silver nanoparticles' FT − IR spectrum after FT − IR spectroscopy was conducted, as illustrated in Figure 6. The N − H stretching vibration, O − H stretching vibration, and organic moiety such as carboxylic acid cause an intense broad band at 3400 cm^−1^. There is correspondence between polysaccharide-oriented aromatic C − C stretching vibrational modes and anionic carboxylate group and the absorption band at 1660 cm^−1^ [81]. This is in good agreement with the report done by Alzahrani et al. [52, 82]. The nanoparticles gain further stability when functionalization of silver nanoparticles that herbal extracts synthesized is undertaken using polysaccharides and aromatic compounds as documented within the FT-IR analysis. Silver ions within silver nanoparticles may be caused by aromatic compounds that exist within plant extracts.

### 3.3. Colorimetric Sensor

Metallic ion forms could accidentally be released when metals are extensively used in different fields [83]. Water bodies and the environment face pollution from such metal ions. Owing to its existence in different forms including organic, inorganic, and elemental, mercury is regarded as the most toxic of all listed pollutants [84]. Conventional systems of detecting metal ions could be considered labour intensive or expensive [85]. The silver nanoparticles' SPR properties to detect transition metal ions, alkaline metal ions, alkali metal ions, and mercury ions were explored in this paper.

#### 3.3.1. Detection of Alkali, Alkaline, and Trivalent Metal Ions

As Section 2.5 describes, 2 mL silver nanoparticle solution was added to 10^−3^ mol L^−1^ and 1 mL concentration containing salt stock solution to investigate how green-synthesized silver nanoparticles react to alkaline and alkali Earth metals. Afterwards, metal ion solutions were added creating an overall volume of 3 mL to investigate how different metal ions affected the LSPRs band intensity and the UV-Vis absorption spectra that corresponded were documented.

**Figure 7:**
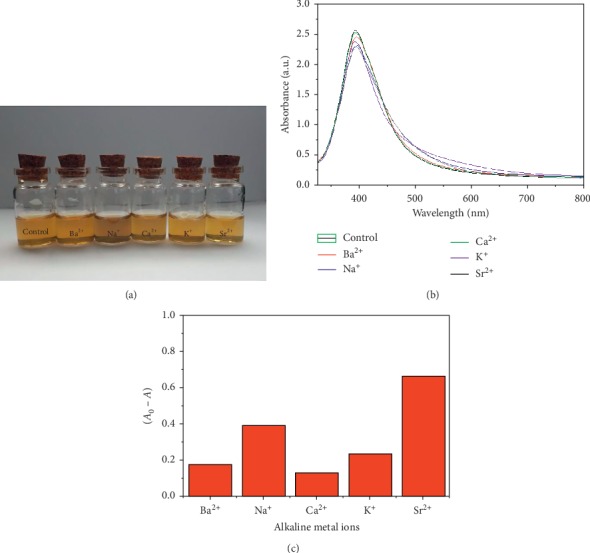
(a) Photo of the green-synthesized AgNPs solution towards various alkali and alkaline metal ions, (b) UV-Vis absorbance of the AgNPs before and after adding 1 mL of different alkali and alkaline metal ions, and (c) absorption ratio (∆*A*) of AgNPs with different metal ions.

Absorption titrations were performed against alkaline metal ions such as Sr^2+^, K^+^, Ca^2+^, Na^+^, and Ba^2+^ ions and alkali metal to investigate the ability of green-fabricated silver nanoparticles to detect these ions. Based on the illustration in Figure 7(a), the silver nanoparticles' absorbance ratio and mixture colour were checked after the interaction of different alkaline metal ions and alkali metals. No change in silver nanoparticle colour was observed when alkaline metal ions or alkali metals were added to silver nanoparticles. The silver nanoparticle solution's UV-Vis absorbance after and before 1 mL of various alkaline metal ions or alkali metals was added and is illustrated in Figure 7(b). A change in spectrum was not observed for alkaline metal ions or alkali metals, and the optimum absorption wavelength of silver nanoparticles was found to be about 405 nm. Changes in colour and LSPR absorption were not observed.

**Figure 8:**
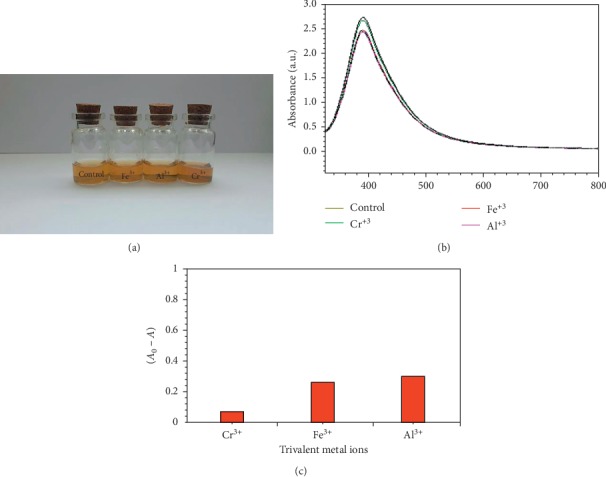
(a) Photo of the green-synthesized AgNPs solution towards various trivalent metal ions, (b) UV-Vis absorbance of the AgNPs before and after adding 1 mL of different trivalent metal ions, and (c) absorption ratio (∆*A*) of AgNPs with various trivalent metal ions.

The colorimetric reaction of silver nanoparticles to different alkaline metal ions and alkali metals is illustrated in Figure 7(c). The detector's selectivity for these metal ions with green-fabricated silver nanoparticles was not observed. The same result was obtained when trivalent metal ions (Fe^3+^, Al^3+^, and Cr^3+^) was used to check the ability of the green-fabricated silver nanoparticles to detect these metal ions (Figure 8).

#### 3.3.2. Detection of Transition Metal Ions

**Figure 9:**
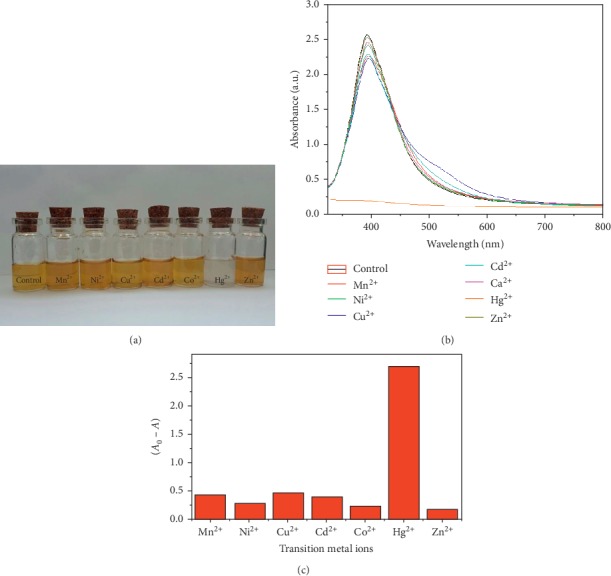
(a) Photo of the green-synthesized AgNPs solution towards various transition metal ions, (b) UV-Vis absorbance of the AgNPs before and after adding 1 mL of different transition metal ions, and (c) absorption ratio (∆*A*) of AgNPs with various transition metal ions.

The study investigated how silver nanoparticle solution was affected by transition metal ions. This was undertaken by examining various heavy metals including Zn^2+^, Hg^2+^, Co^2+^, Cd^2+^, Cu^2+^, Ni^2+^, and Mn^2+^ ions. Unlike other metals, there was a significant colour change in silver nanoparticles for transition metal ions after mercury ions were added, as illustrated in Figure 9(a). The results showed that when mercury ions were added to glass tubes that contained freshly prepared silver nanoparticle solution, there was a colour change for silver nanoparticle solution from brown to transparent; however, for transition heavy metals, there was no change after the silver nanoparticle solution was added, which implies the absence of effect on silver nanoparticle colour. In addition, it was found that the assay method has high specificity and selectivity toward mercury ions and alkaline metal, alkali metal, and other transition metal ions in similar conditions do not experience silver nanoparticle sensitivity.

The silver nanoparticles' UV-Vis absorbance after and before addition of 1 mL of various transition metal ions is illustrated in Figure 9(b). Unlike mercury ions that caused a change in spectrum, the silver nanoparticle colour and LSPR band did not experience any effect; in addition, the maximum wavelength had decreased absorbance, and there was apparition for new LSPR bands at longer UV-Vis spectra wavelength. The optical spectroscopic signatures for closed-shell *d*10 configuration Hg^2+^ do not exist [66]. After mercury was added to silver nanoparticles, solution colour changed to colourless and the silver nanoparticles did not have the LSPR band.

To further study the selective nature of silver nanoparticles towards different metal ions, the silver nanoparticle solution's absorbance was plotted with all transition metal ions. The selective nature of the optimized mercury ion sensor was examined by comparing solutions of other metallic ions against the silver nanoparticle absorbance ratio Δ*A*. The silver nanoparticles Δ*A* intensity ratios of different metal ions clearly showed selectivity for mercury II ions, as illustrated in Figure 9(c). All the observations and results obtained indicate that aggregates of silver nanoparticles whose colour was transparent accounted for a significant ratio, whereas good dispersal of silver nanoparticles was demonstrated by a lower ratio [86]. By comparing Figures 7(c) and 8(c) with Figure 9(c), it is obvious that the absorption ratio (Δ*A* of the green-fabricated silver nanoparticles with different metals (alkali, alkaline, trivalent, and transition metal ions) was less than 1.0 a.u. except for mercury, which was more than 2.5 a.u. These findings showed that silver nanoparticles had a reactive selectivity towards mercury II ions, as the dramatic absorbance ratio increased.

#### 3.3.3. Investigation of Different Volumes of Mercury Ions

**Figure 10:**
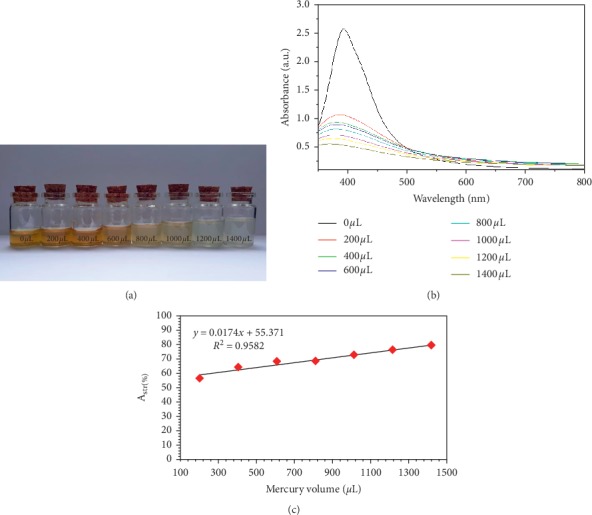
Colorimetric sensing of Hg^2+^ ion: (a) digital photograph, (b) UV-Vis absorption spectra of AgNPs before and after adding different volume of HgCl_2_ solution for 30 seconds, and (c) the function curve of the absorbance value versus the volume of Hg^2+^ in the range of 200–1400 *μ*L at pH 7.2.

To examine the sensitive nature of the method and the minimum detectable mercury II ion within aqueous solutions by monitoring the values of UV-Vis absorbance and the system's colour change, different volumes of mercury ions' aqueous solution were incorporated to 2 mL silver nanoparticle solution in room temperatures. UV-Vis spectra for the silver nanoparticle LSPR property was used to determine the mercury II ion detection limit. There was a change in colour from yellow, light yellow, and light salmon to clear/transparent after different volumes of mercury II solution was introduced incrementally from 0 to 1400 µL, as illustrated in the photograph for silver nanoparticle solution in Figure 10(a). Notably, the trend is significantly attributed to silver nanoparticle oxidation by mercury II ions caused by greater mercury/mercury ion potential (0.851 V), as opposed to silver/silver ions (0.799 V) [52, 87]. The reduction of silver nanoparticle LSPR band intensity was visible with mercury II ions. Because of this, a redox reaction will occur between mercury and silver ions. An amalgam of mercury and silver is formed when mercury II ions are reduced to mercury by the electrochemical reduction potential difference from oxidized silver nanoparticles [85].

Because of the colour change for silver nanoparticle solution, a UV-Vis spectrometer could be used for monitoring the change in LSPR optical properties.  Figure 10(b) illustrates the correlation between mercury II ion and changes in the absorbance strength of LSPR. It could be inferred that the strength of LSPR absorbance changed significantly, and this was dependent on mercury II ion volume. Based on the figure, it could be stated that when mercury II ion increased, the absorbance peak of silver nanoparticles decreased. In addition, the absorption band of the surface plasmon might experience a slight change of blue shift when mercury II ions are increased. Findings have shown that reduction of mercury ions within aqueous silver solution from mercury layers around particles of silver could be undertaken radiolytically, followed by plasmon absorption band's blue shift and broadening [88]. Based on conducted experiments, metallic mercury was formed after silver nanoparticles reacted with the mercury II ion. The silver surface could offer the platform for strong bonding of newly generated mercury atoms, and this might be accounted for by slight-blue changes of the silver nanoparticles' LSPR band. The results could be considered the initial stage in the investigation of mercury ion detection. Indeed, a digestive technique can be used for transforming different forms of mercury such as CH_3_HgCl, CH_3_Hg^+^, HgO, Hg, and Hg(OH)_2_ [89]. In view of this, the proposed study might provide a significant promise as the method for colorimetrically detecting overall forms of mercury.

Figure 10(c) illustrates the plot for colorimetric response (*A*_str_) against the mercury II ion volume through a construction of the absorption spectra that produced the calibration curve (*A*_str_ vs Hg^2+^ volume). A linear correlation (*y*=0.0174*x*+55.371, *R*^2^=0.9582), between the mercury II ion volume and absorbance intensity, changes in the range of 0 µL to 1400 µL at 405 nm. In view of this, it could be inferred that mercury II ion could be detected colorimetrically using green-synthesized silver nanoparticles. Sakly et al. [53] fabricated AgNPs coated with carboxymethyl cellulose (CMC-capped AgNPs) from date palm tree for Hg^2+^ ion detection with 95% of good recovery, and RSD does not exceed 8%. Although the recovery in this study is slightly low (92%) compared with the previous study, the detection method was fast, simple, and selective colorimetric assay for Hg(II) ions. In addition, the procedure for fabrication of green AgNPs using onion extract was simple and fast.

**Figure 11:**
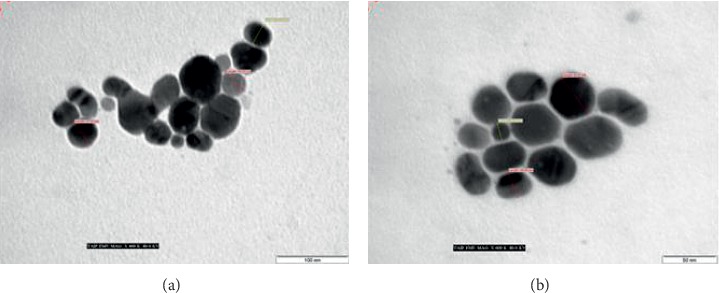
TEM micrographs of AgNPs after introducing 400 *μ*L of mercury (II) ion.

After addition of mercury II ions, TEM analysis was used for examining the fabricated silver nanoparticles. Contrary to the results in Figures 2 and 11 showed that the introduction of mercury II ions completely changed the silver nanoparticles' morphology. The absorbance of the LSPR band had effective excitation, which was attributed to the dispersion media having dispersed and stable silver nanoparticles prior to addition of mercury II ions. In contrast, the introduction of mercury II ions to silver nanoparticles caused aggregation of nanoparticles through catalytic reactions between Hg^2+^ ions and silver nanoparticles; notably, the nanoparticle aggregation decreased the absorbance strength of the LSPR band. In addition, silver nanoparticle aggregation did not exist alongside other metal ions.

### 3.4. Practical Application

Notably, real samples of water were used at the micromolar level for evaluating the feasibility of the proposed detection method for mercury II ions. A specific volume obtained from the sample with drinking water was added to as-prepared colloidal solution that was spiked initially using NaCl aqueous solution (overall concentration 250 mM) before each sample was loaded with toxic ion. There was no detection of LSPR absorption spectra, regardless of the composition. Different mercury II ions mixed with spiked samples of drinking water were used for performing recovery experiments. Based on the results in Table 1, a relative standard deviation (RSD) of below 6% and suitable recovery of over 92% were obtained. In addition, the table indicates that mercury II ion detection was not interfered with the composition of water samples. Besides, measurement repeatability was conducted on 3 colloidal solution replicates under similar experimental conditions. Because the RSD does surpass 6%, the proposed assay could be considered reliable as confirmed by the results. Moreover, consistent with results, mercury poisoning could be prevented by potentially applying the proposed colorimetric assay.

## 4. Conclusion

Overall, a selective, highly sensitive, rapid, ecofriendly, cost-effective, label-free, and simple colorimetric assay to detect mercury II ions in samples of drinking water using green-prepared silver nanoparticles and onion extracts as the colorimetric probe, which allow mercury II ions to be detected in real time and rapidly, has been developed. Good stability and localized surface plasmons are exhibited by the green-synthesized silver nanoparticles. A similar chemistry is used for silver nanoparticle lowering LSPRs to etch out silver ions during mercury II ion sensing. Selective, rapid, cost-effective, and simple mercury II ion sensing within real water and biological samples in the future will benefit from this chemistry. Accordingly, plasmonic-assisted colorimetric assays for detecting mercury II ion will be enhanced when methodologies are enriched, and new approaches were applied through successful and full implementation of the eco-friendly colorimetric assay. Currently, the focus is on developing highly sensitive and selective (up to picomolar or nanomolar concentration range) for detection of hazardous metal ions using silver nanoparticles stabilized using onion extracts.

## Figures and Tables

**Table 1:** Estimation of Hg^2+^ concentration in real water samples using the fabricated SNPs as a sensor.

Sample	Spiked (*μ*M)	Recovery (%)	RSD (%) *n* = 3
Ground water	300	97.8	5.97
	600	98. 6	4.82
	900	93.5	3.84

Tap water	300	95.2	5.88
	600	97.4	4.76
	900	92.1	1.59

RSD = relative standard deviation.

## Data Availability

The data used to support the findings of this study are included within the article.
